# The effect of metformin on senescence of T lymphocytes

**DOI:** 10.1186/s12979-023-00394-0

**Published:** 2023-12-12

**Authors:** Jia Yang, Hai-Cheng Liu, Jian-Qing Zhang, Jian-Yong Zou, Xin Zhang, Wo-Ming Chen, Yong Gu, Hai Hong

**Affiliations:** 1 The First Affiliated Hospital of Sun Yat-Sen University, No.58 Zhong Shan Two Road, Guang Zhou, 510000 Guang Dong China; 2 Key Laboratory of Tropical Disease Control of Sun Yat-Sen University, Ministry of Education, The Institute of Immunology of Zhong Shan Medical School, Sun Yat-Sen University, No.74 Zhong Shan Two Road, Guang Zhou, Guang Dong 510000 China; 3 Sun Yat-Sen Memorial Hospital, Sun Yat-Sen University, 107 Yan Jiang West Road, Guang Zhou, 510120 China

**Keywords:** Senescent T cell, Metformin, Inhibition, SASP

## Abstract

**Background:**

Immunosenescence occurs as people age, leading to an increased incidence of age-related diseases. The number of senescent T cells also rises with age. T cell senescence and immune response dysfunction can result in a decline in immune function, especially in anti-tumor immune responses. Metformin has been shown to have various beneficial effects on health, such as lowering blood sugar levels, reducing the risk of cancer development, and slowing down the aging process. However, the immunomodulatory effects of metformin on senescent T cells still need to be investigated.

**Methods:**

PBMCs isolation from different age population (*n* = 88); Flow Cytometry is applied to determine the phenotypic characterization of senescent T lymphocytes; intracellular staining is applied to determine the function of senescent T cells; Enzyme-Linked Immunosorbent Assay (ELISA) is employed to test the telomerase concentration. The RNA-seq analysis of gene expression associated with T cell senescence.

**Results:**

The middle-aged group had the highest proportion of senescent T cells. We found that metformin could decrease the number of CD8 + senescent T cells. Metformin affects the secretion of SASP, inhibiting the secretion of IFN-γ in CD8 + senescent T cells. Furthermore, metformin treatment restrained the production of the proinflammatory cytokine IL-6 in lymphocytes. Metformin had minimal effects on Granzyme B secretion in senescent T cells, but it promoted the production of TNF-α in senescent T cells. Additionally, metformin increased the concentration of telomerase and the frequency of undifferentiated T cells. The results of RNA-seq showed that metformin promoted the expression of genes related to stemness and telomerase activity, while inhibiting the expression of DNA damage-associated genes.

**Conclusion:**

Our findings reveal that metformin could inhibit T cell senescence in terms of cell number, effector function, telomerase content and gene expression in middle-aged individuals, which may serve as a promising approach for preventing age-related diseases in this population.

**Supplementary Information:**

The online version contains supplementary material available at 10.1186/s12979-023-00394-0.

## Introduction

With the rapid growth of the elderly population, the increasing incidence of age-related diseases has gradually attracted attention. Accumulating evidence suggests that chronic, low-grade inflammation in the elderly population, caused by the dysfunctional immune system's inability to effectively eliminate pathogens, contributes to the development of age-related diseases such as malignant tumors, endocrine and metabolic diseases, Alzheimer's disease, atherosclerosis, and autoimmune diseases. This leads to increased morbidity and mortality in the elderly. The age-related decline in immune function is referred to as immunesenescence [1].

T cells play a central role in the immune response, including anti-tumor immunity. As individuals age, the number of senescent T cells increases [2]. Aged T cells show distinct surface markers, such as the loss of expression of costimulatory receptors CD27 and CD28, and high expression of CD57, KLRG-1, CD45RA. Additionally, chemokine receptors CCR7 are down-regulated in these senescent T cells, which are located in the effector T cell population [3–5]. Cellular senescence causes chronic inflammation through the senescence-associated secretory phenotype (SASP). Senescent T cells have high levels of intracellular granules and secrete inflammatory cytokines, such as IFN-γ and TNF-α, after short-term activation [6, 7]. T cell senescence is associated with low proliferative activity due to the loss of telomerase activity and telomere shortening [8, 9]. Some epidemiological studies have found an increase in telomere shortening in individuals with age-related diseases [10]. Senescent T cells with the shortest telomeres are usually found within the CD27-CD28- population [11]. These T cells display multiple characteristics of senescence, including high levels of ROS, low proliferative activity, DNA damage, and cell cycle arrest [9, 12].

Metformin is a first-line drug for the treatment of type 2 diabetes. It exhibits hypoglycemic effects and has been proven to have additional properties like anti-cancer effects and the prevention and treatment of aging-related disorders [13, 14], as well as the inhibition of inflammaging [15]. While the role of metformin in the immune system has received significant attention, little is known about its immunomodulatory effects on senescent T cells.

In this study, we observed that the middle-aged subject had the highest proportion of senescent T cells among different age groups. We found that metformin could decrease the number of CD8 + senescent T cells. Additionally, metformin affects the secretion of SASP, inhibiting the production of IFN-γ in CD8 + senescent T cells. Metformin treatment restrained the production of the proinflammatory cytokine IL-6 in lymphocytes. Metformin had minimal effects on Granzyme B secretion in senescent T cells, it promoted the production of TNF-α in senescent T cells. Furthermore, metformin increased the concentration of telomerase and the frequency of undifferentiated T cells. The results of RNA-seq showed that metformin promoted the expression of genes related to stemness and telomerase activity, while inhibiting the expression of DNA damage-associated genes. Overall, our findings reveal that metformin could inhibit T cell senescence in terms of cell number, effector function, telomerase content and gene expression in middle-aged individuals, which may serve as a promising approach for preventing age-related diseases in this population.

## Results

### The highest frequency of CD8 + senescent T cells in middle-age population

Age-related increases in senescent cells were previously believed to be associated with increased susceptibility to tumors, infectious diseases, and poor responses to vaccination [16]. In fact, middle-aged people are thought to be more likely to experience chronic fatigue under multiple stresses, which can easily induce inflammatory states and abnormal immune responses to antigens [17]. We analyzed clinical samples from Sun Yat-sen University First Affiliated Hospital over the past six years and found that the proportion of lung cancer patients increased significantly in the middle-aged age group compared to the young and elderly groups (Table 1) (Supplementary Fig. 1a). The distribution of senescent T cells in the middle-aged population is still unclear.

**Table 1:** Lung cancer patient samples from The First Affiliated Hospital of Sun Yat-sen University over the past six years (*n* = 4498)

Variable	Male	Female	Total	p^*^
	**N**	**N**	**N**	
Age				
< 45	205	381	586	< 0.0001
45–65	1206	1524	2730	
> 65	630	552	1182	

^*^Ordinary one-way ANOVA test; Level of significance: *p* < 0.05

**Fig. 1:**
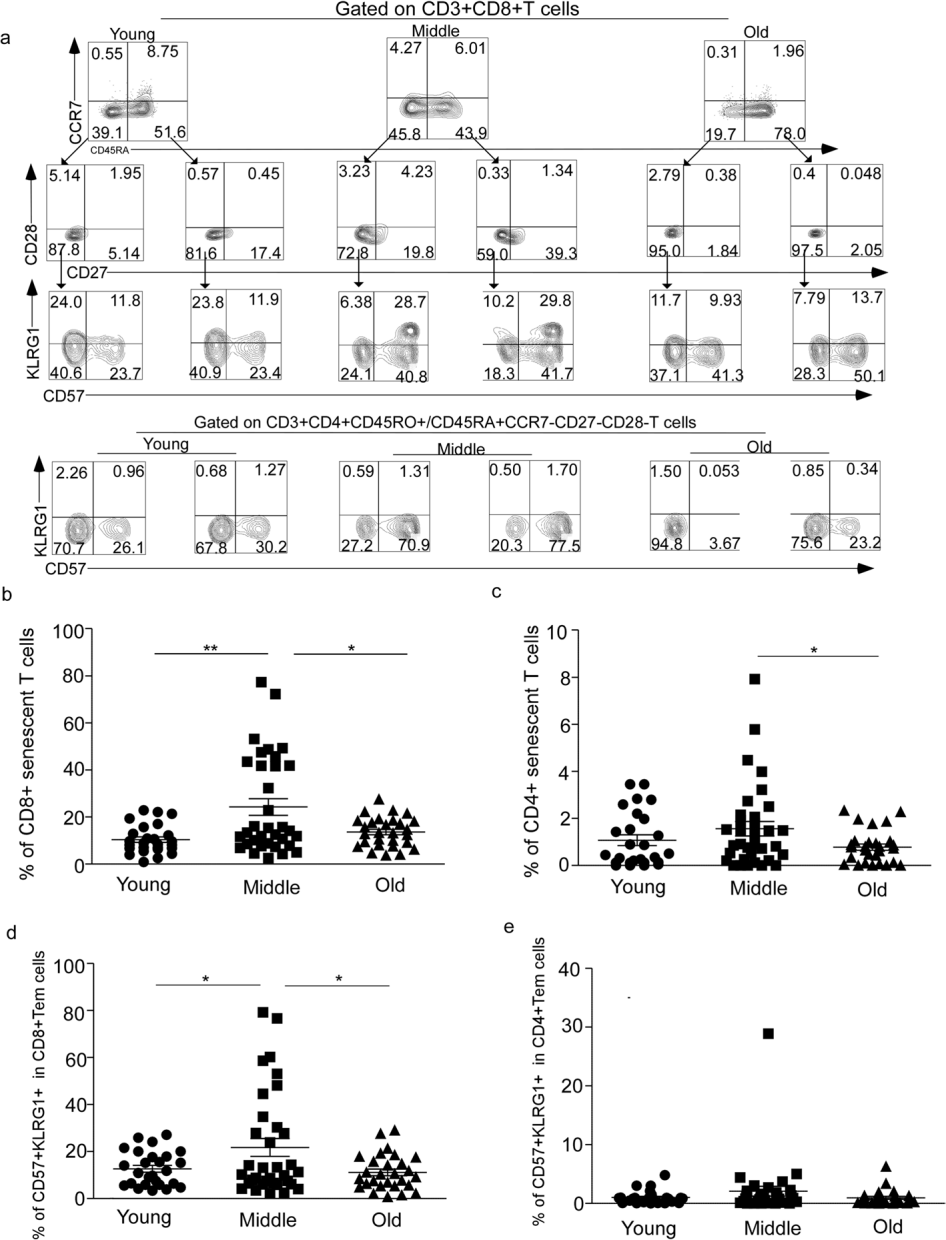
The highest frequency of CD8 + senescent T cells in middle-age population was analyzed by FACS. **a** Representative flow cytometric analyses of senescent T cells (CD3 + CD45RA + CCR7-CD27-CD28-CD57 + KLRG1 +) in CD8 and CD4 T cells (top quadrant) and representative flow cytometric analyses of CD27-CD28-CD57 + KLRG1 + subsets, gated on CD3 + CD4 + /CD8 + CD45RO + CCR7-(Tem) population (bottom quadrant). The frequency of CD8 + senescent T cells (**b**) and CD4 + senescent T cells (**c**) at young group, middle-age group and elderly group. The percentage of CD57 + KLRG1 + expression on CD3 + CD8 + CD45RO + CCR7-CD27-CD28-population (**d**) and CD3 + CD4 + CD45RO + CCR7-CD27-CD28-population (**e**) at different age groups. Expressed as the mean ± SEM. *P < 0.05, **P < 0.01; Mann– Whitney test (two-tailed) and nonpaired Student’s t-test. Tem, effector memory T cell

To determine the difference in the number of senescent T cells in the middle-aged group compared to the young and elderly groups, we recruited 88 healthy donors, PBMCs from the 88 donors between the ages of 26 and 86 years, we divided our donors into three groups, young group (< 45 years old,mean age = 34.1), middle-age group (45–65 years old,mean age = 54.13) and elderly group (> 65 years old,mean age = 75.57) [18]. All donors were HIV negative and free of cancer and other infection diseases. Mononuclear lymphocytes were shown by representative flow cytometry analysis of CD3 + CD8 + /CD4 + CD45RA + CCR7-CD27-CD28-CD57 + KLRG1 + expression indicating frequency of senescent T cells (Fig. 1a); The frequency of CD8 + senescent T cells (24.26 ± 3.563, *N* = 34) was significant increase in middle-aged group compare to the young group (10.36 ± 1.186, *N* = 26) and elderly group( 13.64 ± 1.177, *N* = 28) (*p* = 0.017, *p* = 0.0115) (Fig. 1b), similar results were observed in CD4 + senescent T cell subsets (*p* = 0.0329,Fig. 1c) ( young:1.075 ± 0.2275 *N* = 26; middle:1.564 ± 0.3059 *N* = 34;elderly: 0.7812 ± 0.1327 *N* = 28) and the proportion of CD8 + senescent T cells was higher than that of CD4 + T cell at each age group (*p* < 0.001, Supplementary Fig. 1b). Senescent T cell originated from effector T cells (Teff) which were at the end stage of T cell differentiation. We observed that middle-aged people had the most abundant effector T cells in CD8 + T cell population (Supplementary Fig. 1c). Additionally, we found that the frequency of CD8 + Teff cells was higher than that of CD4 + Teff cells at different age group (*p* < 0.05, *p* < 0.001, Supplementary Fig. 1d).

As the thymus gradually shrinks with age, the number of naïve T cells decrease while memory T cells increase [19]. Based on the differentiation pattern of T cells in peripheral lymphoid organs as against the antigen, memory T cells differentiated into effector T cells [20]. The composition of CD27-CD28-CD57 + KLRG1 + subsets in CD3 + CD45RO + /CD45RA-CCR7- population was analyzed to determine whether the precursors of senescent T cells are present in effector memory T cell (Tem) cells (Fig. 1a). It was found that although the proportion of Tem cells was highest in the elderly group (Supplementary Fig. 1c), the frequency of CD57 + KLRG1 + double positive cells from CD8 + CD45RO + /CD45RA-CCR7-CD27-CD28- was still the highest in the middle-aged group (young: 12.66 ± 1.439 *N* = 26; middle: 21.74 ± 3.794 *N* = 34; elderly: 11.15 ± 1.422 *N* = 28) (*p* = 0.0494, *p* = 0.0187, Fig. 1d). The proportion of CD4 + CD45RO + /CD45RA-CCR7-CD27-CD28-CD57 + KLRG1 + T cells was roughly the same across the different age groups (Fig. 1e). Additionally, more CD57 + KLRG1 + subsets were observed in CD8 + T cells than in CD4 + T cells within the Tem subsets (Fig. 1d, e). However, the number of CD4 + Tem was higher than that of CD8 + Tem across age groups (*p* < 0.05, *p* < 0.001, Supplementary Fig. 1e).

These results indicate that the frequency of senescent T cells was highest in the middle-aged group, and the proportion of CD8 + senescent T cells was higher than that of CD4 + senescent T cells. Furthermore, the proportion of Tem cells with senescent T cell phenotype was highest in the middle-aged group.

### The number of senescent T cells significantly reduced with Metformin treatment

**Fig. 2:**
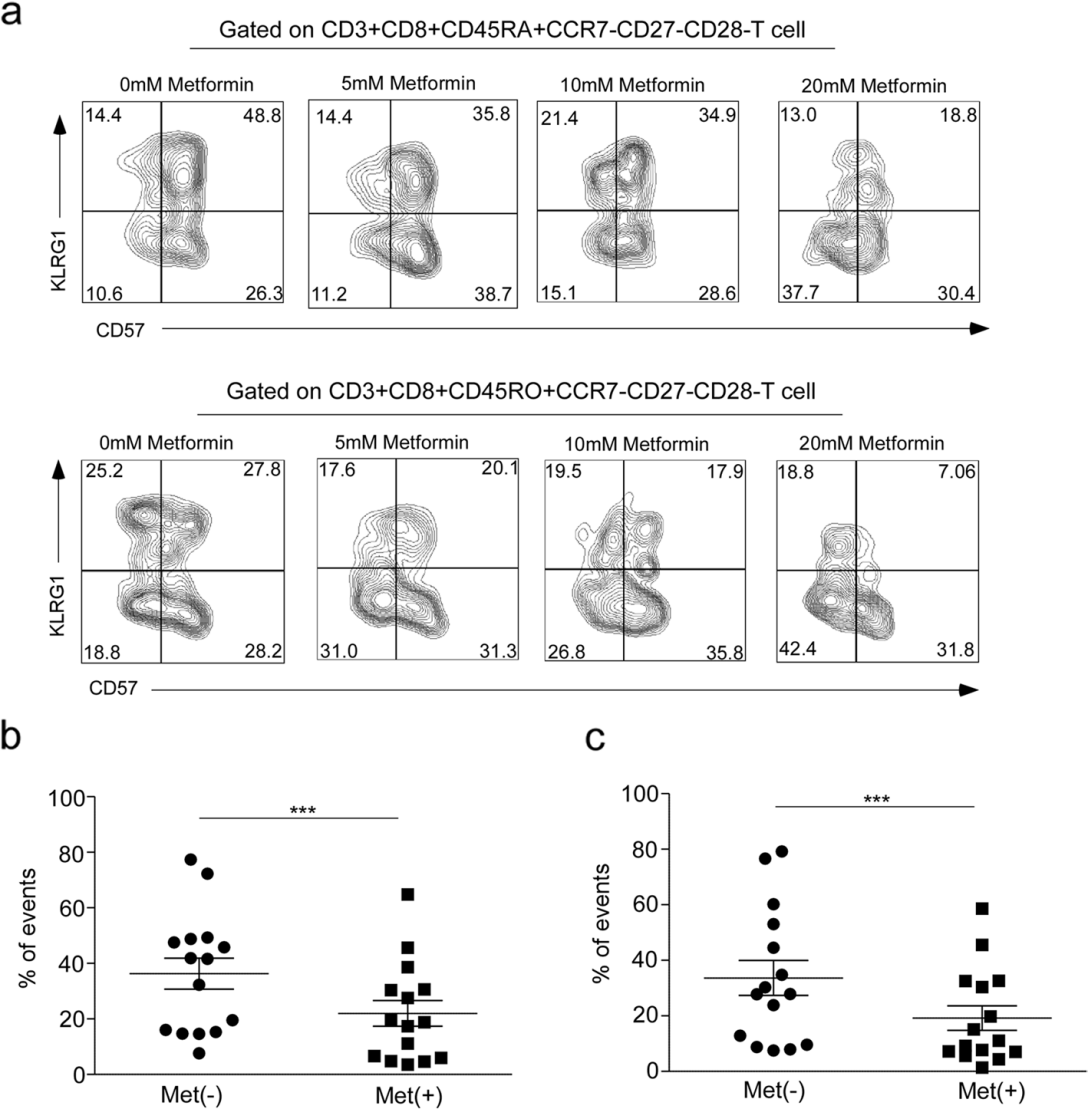
The number of CD8 + senescent T cells in middle**-**age group decreased significantly after metformin treatment. (**a**) Frequency of CD3 + CD8 + CD45RA + /CD45RO + CCR7-CD27-CD28- cells which gated on CD3 + CD8 + T cells treated with the different concentrations of metformin (0 mM, 5 mM, 10 mM or 20 mM). Quantification of the percentage of CD8 + senescent T cells derived from Teff cells (CD3 + CD8 + CD45RA + CCR7-CD27-CD28-CD57 + KLRG1 +) (**b**) and Tem cells (CD8 + CDR45RO + CCR7-CD27-CD28-CD57 + KLRG1 +) (**c**) at middle-age group with 20 mM metformin treatment. Expressed as the mean ± SEM. ***P < 0.001; Mann–Whitney test (two-tailed) and paired Student’s t-test. Teff, effector T cells; Tem, effector memory T cells; Met(-), control; Met( +), 20 mM metformin treatment

It is important to reduce senescent T cells through interventions to prevent the occurrence of age-related diseases, especially tumors. Metformin not only has a hypoglycemic effect but also plays a role in anti-tumor and anti-aging [21, 22]. There is little evidence supporting the modulation of T cell senescence by metformin. To investigate the effect of metformin on senescent T cells, PBMCs derived from a middle-aged population were treated with different concentrations of metformin in vitro: 0 mM, 5 mM, 10 mM, and 20 mM. The results showed that treatment with 5 mM metformin had no significant effect on senescent T cells (Supplementary Fig. 2a). The number of senescent T cells significantly decreased after treatment with 10 mM metformin (from 47.29% to 34.3%, with a 27.5% inhibition rate) (*p* < 0.001, Supplementary Fig. 2a). The frequency of CD8 + senescent T cells also decreased with 20 mM metformin treatment (from 36.3% to 22%, with a 39.4% inhibition rate) (*p* = 0.004, Fig. 2a, b). Therefore, a metformin concentration of 20 mM was determined for the subsequent research. Similar inhibition was observed in subsets of CD8 + CD45RO + CCR7-CD27-CD28-CD57 + KLRG1 + cells from Tem cells (*p* = 0.0005, Fig. 2a, c, Supplementary Fig. 2b). Our findings show that metformin could reduce the frequency of senescent T cells in middle-aged populations.

### Metformin reduces the secretion of IFN-γ in CD8 + senescent T cells

**Fig. 3:**
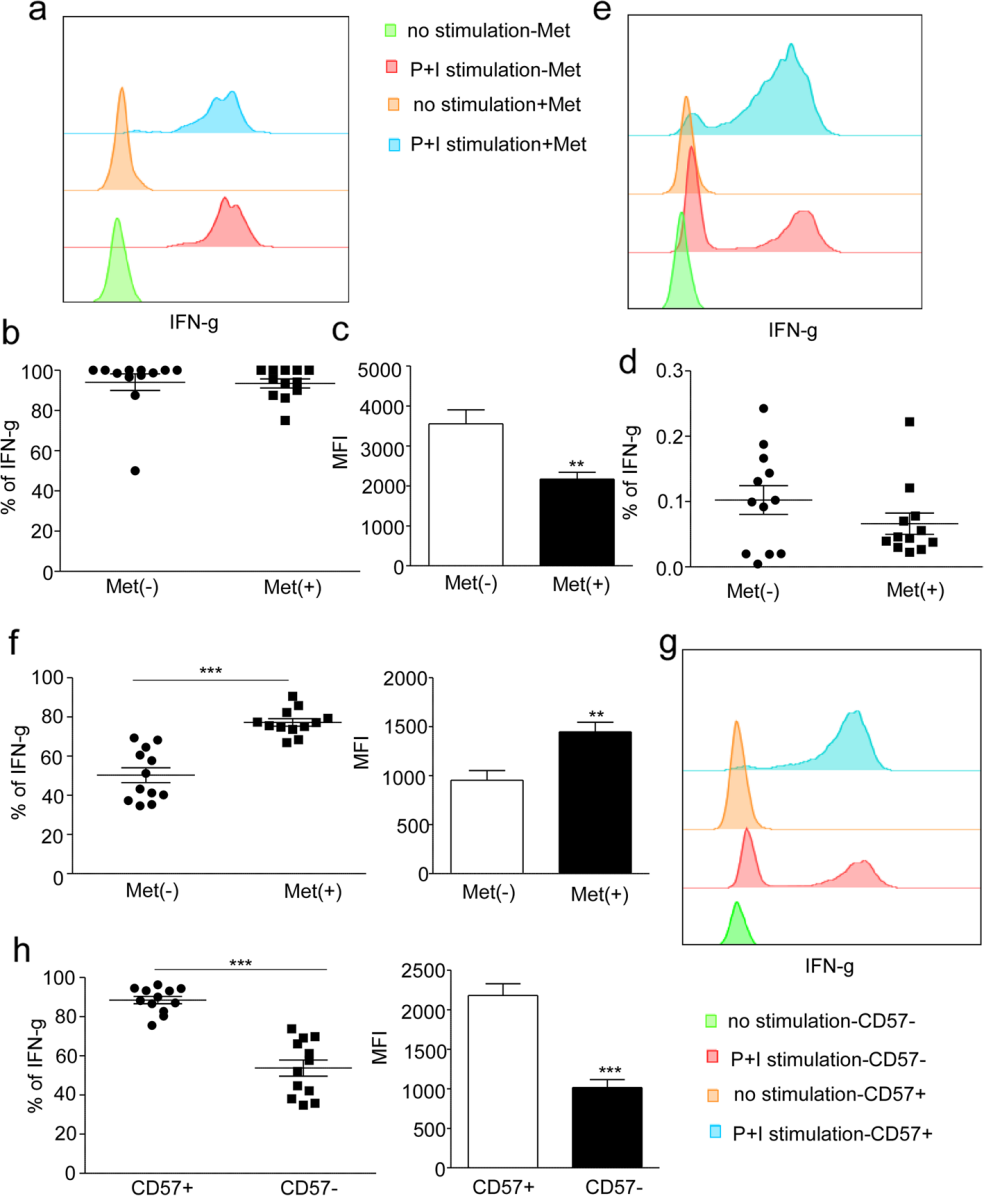
Metformin reduces the secretion of IFN-γ in CD8 + senescent T cells. Representative flow histogram of IFN-γ production by CD8 + senescent T cells (CD3 + CD8 + CD45RA + CCR7-CD27-CD28-CD57 + KLRG1 +) (**a**) and non- senescent T cells (CD3 + CD8 + CD45RA + CCR7-CD27-CD28-CD57-KLRG1-) (**e**) in the control group and the 20 mM Met treatment group relative to the unstimulated controls from middle-age subjects. Analysis of IFN-γ-secreting CD8 + senescent T cells (**b**) and MFI of IFN-γ in CD8 + senescent T cells (**c**) with 20 mM metformin treatment. **d** Frequency of CD3 + CD8 + CD45RA + CCR7-CD27-CD28-CD57 + KLRG1 + IFN-γ + T cells in lymphocytes with Met treatment and control. **f** Percentage and MFI of IFN-γ in CD3 + CD8 + CD45RA + CCR7-CD27-CD28-CD57-KLRG1- T cells with Met treatment and control. **g** Representative flow histogram of IFN-γ secretion by CD3 + CD8 + CD45RA + CCR7-CD27-CD28-CD57 + /CD57- population relative to the unstimulated controls from middle-age donors. **h** Percentage and MFI of IFN-γ in CD3 + CD8 + CD45RA + CCR7-CD27-CD28-CD57 + / CD57- population. Expressed as the mean ± SEM. **P < 0.01, ***P < 0.001; Paired t test. Met, metformin

SASP (senescence-associated secretory phenotype, SASP) secreted by senescent T cells could be harmful to neighboring healthy cells and even induce senescence [7]. IFN-γ is highly expressed in senescent T cells [23]. To investigate whether metformin inhibits the production of IFN-γ, PBMCs from the middle-aged group were treated with 20 mM metformin for 24 h. There was no significant difference in the production of IFN-γ from CD8 + senescent T cells between the metformin treatment and control groups (Fig. 3a, b); however, compared with the control group, the average fluorescence intensity (MFI) of IFN-γ in CD8 + senescent T cells in the treatment group was significantly decreased (*P* < 0.01, Fig. 3c). Moreover, the frequency of CD3 + CD8 + CD45RA + CCR7-CD27- CD28- CD57 + KLRG1 + IFN-γ + T cells in lymphocytes decreased after metformin treatment (*p* = 0.064, Fig. 3d). For non-senescent T cells (CD3 + CD8 + CD45RA + CCR7-CD27-CD28-CD57-KLRG1-), the production of IFN-γ increased with metformin treatment (*P* < 0.001, Fig. 3e, f). Similar results were observed in CD4 + T cells (Supplementary Fig. 3a, b).

Studies have revealed that capacity of proliferation on CD57 + T cells was seriously impaired, and CD57 is considered as the best marker of cellular senescence [24]. We analyzed the differences between CD57- and CD57 + T cell subsets. More IFN-γ production in CD3 + CD8 + CD45RA + CCR7-CD27-CD28-CD57 + T cells than in CD3 + CD8 + CD45RA + CCR7-CD27-CD28-CD57-T cells was observed (*P* < 0.001, Fig. 3g, h).

Together, these results indicate that metformin inhibits the secretion of IFN-γ in senescent T cells but does not down-regulate IFN-γ production in non-senescent T cells.

### Metformin inhibit proinflammatory cytokine IL-6 production

**Fig. 4:**
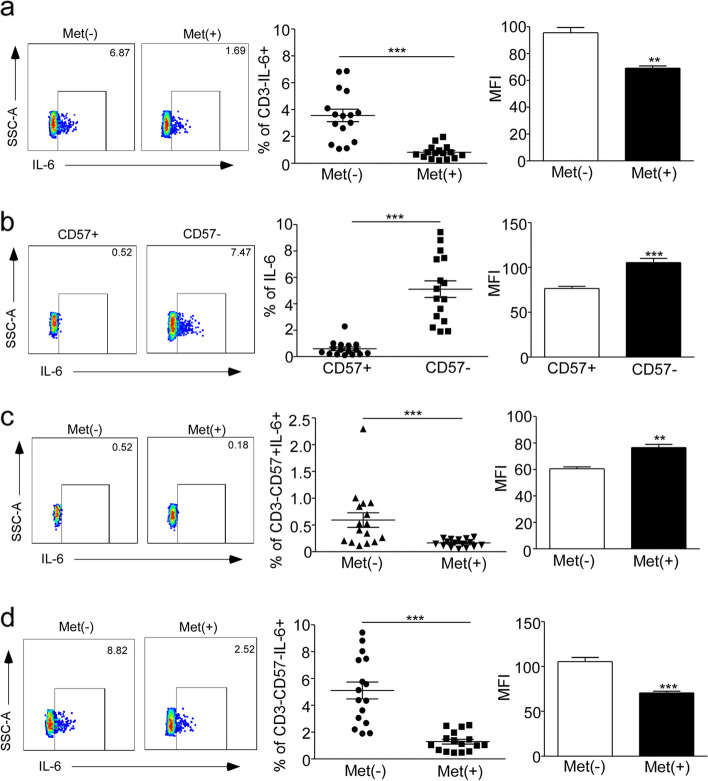
Metformin inhibited the production of proinflammatory cytokine IL-6 in CD3 negative cells. Representative flow analysis of IL-6 production and MFI level in CD3- cells (**a**), CD3-CD57 + cells (**c**) and CD3-CD57- cells (**d**) between 20 mM Met treatment and control for 24 h in middle age group. Representative flow analysis of IL-6 production and MFI in CD3-CD57 + and CD3-CD57- population (**b**). Expressed as the mean ± SEM. ***P < 0.001; Paired *t* test. Met, metformin

IL-6 is one of the SASP factors that are present in various types of senescent cells during aging [25]. This cytokine is primarily secreted by innate immune cells, such as monocytes and dendritic cells (DC) [26]. To identify whether metformin inhibit IL-6 production, we analyzed the secretion of IL-6 in CD3-negative cells from the peripheral blood of middle-aged subjects. The results showed that the percentage of IL-6 producers in the control group was 3.8% in CD3-cells, whereas it was only 1% in CD3-cells with metformin treatment. Additionally, the levels of IL-6 expression (MFI) in CD3- cells were significantly decreased upon metformin treatment (*P* < 0.001, Fig. 4a). We also compared the frequency of IL-6-producing CD3-CD57- T cells with that of CD3-CD57 + T cells, and found that the former had a higher frequency (*P* < 0.001, Fig. 4b). CD57, recognized as a surface marker of replicative senescence in T cells, is also expressed on maturing NK cells [27]. However, it remains unclear whether CD3-CD57 + cells exhibit a cellular senescent as well as CD3 + CD57 + T cells. we examined that the frequencies of IL-6-producing cells were reduced in both CD3-CD57 + cells (*P* < 0.001, Fig. 4c) and CD3-CD57- cells with metformin treatment compared to the control group (*P* < 0.001, Fig. 4d). Similarly, a small proportion of IL-6-producing cells was observed in CD3 + CD8 + /CD4 + T cells, and metformin treatment led to a decrease in the levels of IL-6 production. This consistent decrease was also observed at the MFI levels of IL-6 (*P* < 0.05, Supplementary Fig. 4a, b).

Taken together, our functional analysis suggests that CD3-CD57- cells have a greater capacity to produce IL-6 than CD3-CD57 + cells, and that metformin reduces IL-6 production in both CD3- cells and T cells.

### Metformin has little impact on Granzyme B secretion in senescent T cells

**Fig. 5:**
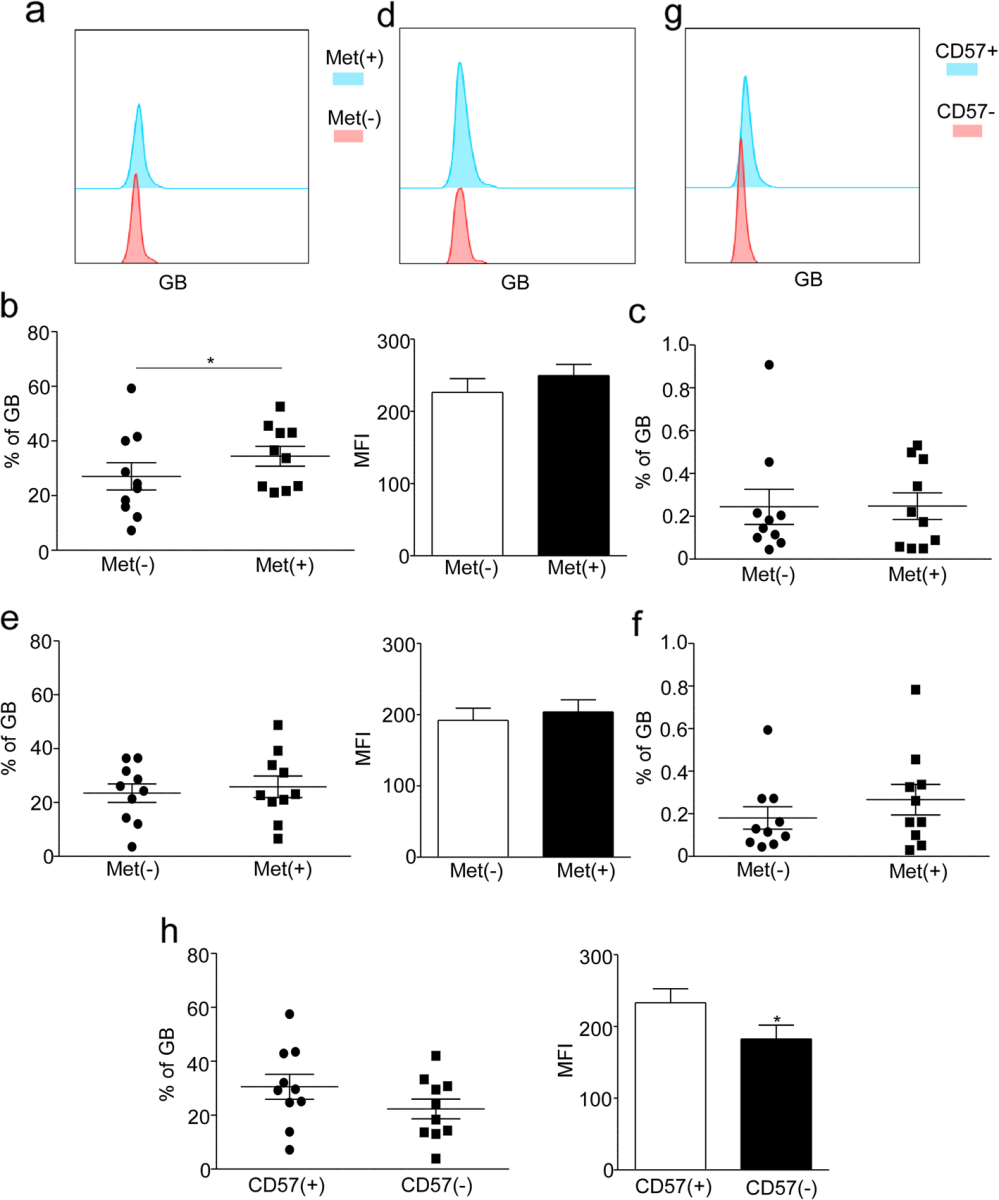
The effect of Metformin on GB secretion. Representative flow histogram of GB secretion by CD3 + CD8 + CD45RA + CCR7-CD27-CD28-CD57 + KLRG1 + (**a**) and CD3 + CD8 + CD45RA + CCR7-CD27-CD28-CD57-KLRG1- (**d**) in the control group and the 20 mM Met treatment group relative to the unstimulated controls group from middle-age donors. Analysis of GB production in CD3 + CD8 + CD45RA + CCR7-CD27-CD28-CD57 + KLRG1 + (**b**) / CD57-KLRG1- (**e**) T cells, cultured with 0 mM or 20 mM metformin for 24 h in the middle age group. The proportion of CD3 + CD8 + CD45RA + CCR7-CD27-CD28-CD57 + KLRG1 + GB + (**c**) and CD57-KLRG1-GB + (**f**) T cells in lymphocytes with metformin treatment compare to control group. **g** Representative flow histogram of GB secretion by CD3 + CD57 + and CD3 + CD57-T cells relative to the unstimulated controls group in middle-age donors. **h** Percentage and MFI of GB in CD3 + CD57 + and CD3 + CD57-T cells. Expressed as the mean ± SEM. *P < 0.05; Paired *t* test. Met, metformin

Cytotoxic T cells (CTLs) and Natural Killer cells (NK) are responsible for producing Granzyme B (GB), which mediates cytotoxicity and kills target cells, senescent T cells express high levels of GB [4]. In this study, we investigated the effect of metformin treatment on GB production. Our findings indicate that metformin treatment leads to an increase in GB production (*P* < 0.05, Fig. 5a, b), while there was no significant difference of MFI (Fig. 5b) and the expression of GB in CD3 + CD8 + CD45RA + CCR7-CD27-CD28-CD57 + KLRG1 + T cells from lymphocytes between the metformin treatment and control groups (Fig. 5c). In non-senescent T cells, no significant differences in GB production were observed with metformin treatment (Fig. 5d, e), although there is an increasing tendency of frequency of CD3 + CD8 + CD45RA + CCR7-CD27-CD28-CD57-KLRG1-GB + T cells in lymphocytes compared to the control group (Fig. 5f). Interestingly, our results demonstrate that CD3 + CD57 + T cells, when compared to CD3 + CD57-T cell populations, secrete higher levels of GB (Fig. 5g, h). Overall, our findings suggest that metformin has little impact on Granzyme B secretion in senescent T cells.

### Metformin promotes the production of TNF-α in senescence T cells

**Fig. 6:**
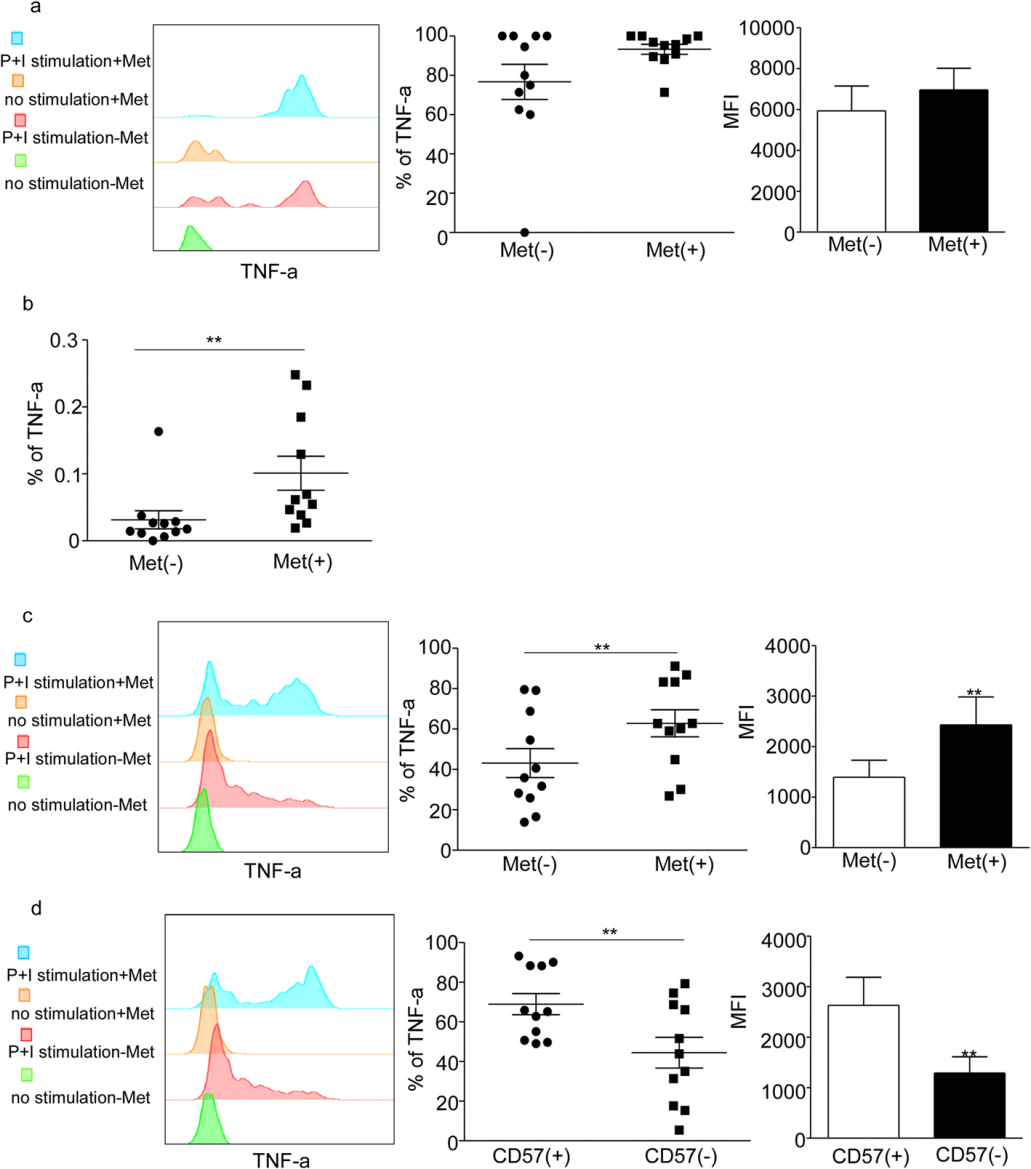
Metformin promotes the production of TNF-α in senescence T cells. Analysis of TNF-α-secreting cells from CD8 + senescent T cells (CD3 + CD8 + CD45RA + CCR7-CD27-CD28-CD57 + KLRG1 +) (**a**) and CD3 + CD8 + CD45RA + CCR7-CD27-CD28-CD57-KLRG1-T cells (**c**) in the control group and the 20 mM Met treatment group in middle-age group. **b** The frequency of TNF-α + CD8 + senescent T cells in lymphocytes between the control and Met treatment group. **d** Representative flow histogram of TNF-α in CD8 + CD45RA + CCR7-CD27-CD28-CD57 + /CD57-T cell and the percentage and MFI of TNF-α in CD8 + CD45RA + CCR7-CD27-CD28-CD57 + /CD57-T cell. Expressed as the mean ± SEM. **P < 0.01; Paired *t* test. Met, metformin

TNF-α, tumor necrosis factor α, is an effective pro-inflammatory cytokine that plays a crucial role in the maintenance and homeostasis of the immune system, inflammation, and host defense. High concentrations of TNF-α can contribute to various pathological processes, including inflammaging, autoimmune diseases, and tumors. It has been observed that senescent T cells also secrete high levels of TNF-α [4, 28, 29]. After intervention with Metformin, an increased tendency in TNF-α secretion from CD8 + senescent T cells was observed when compared to the control group (Fig. 6a). The frequency of CD3 + CD8 + CD45RA + CCR7-CD27-CD28-CD57 + KLRG1 + TNF-α + T cells in lymphocytes also significantly increased (*p* < 0.01, Fig. 6b). Similar results were observed in CD3 + CD8 + CD45RA + CCR7-CD27-CD28-CD57-KLRG1-T cells (*p* < 0.01, Fig. 6c). As expected, CD8 + CD45RA + CCR7-CD27-CD28-CD57 + T cell subsets secreted significantly more TNF-α compared to the CD57- subgroup (*p* < 0.01, Fig. 6d). Additionally, CD4 + senescent T cells showed an increased tendency in TNF-α production with metformin treatment (Supplementary Fig. 5a). However, there was no difference in TNF-α production in CD3 + CD4 + CD45RA + CCR7-CD27-CD28-CD57-KLRG1- T cells with metformin treatment (supplementary Fig. 5b); instead, the frequency of CD3 + CD4 + CD45RA + CCR7-CD27-CD28-CD57-KLRG1-TNF-α + T cells in lymphocytes significantly decreased (*p* < 0.05, Supplementary Fig. 5c). Overall, our results demonstrate that metformin promotes the production of TNF-α in senescent T cells.

### Metformin increased the telomerase concentration and the frequency of undifferentiated T cells

**Fig. 7:**
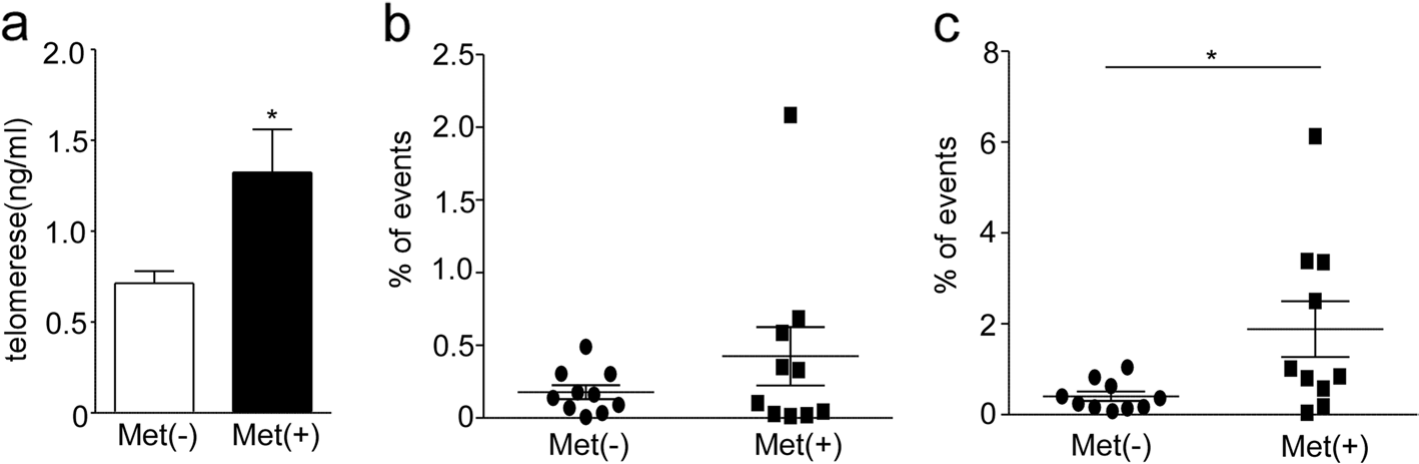
Metformin increased the telomerase concentration and the undifferentiated T cells. **a** The concentration of telomerase in medium (ng/ml) was analyzed in PBMCs with 20 mM Metformin treatment and control at middle age group, examined by ELISA. The flow cytometric analysis the frequency of CD3 + CD4 + CD45RA + CCR7 + CD27 + CD28 + (**b**) and CD3 + CD8 + CD45RA + CCR7 + CD27 + CD28 + (**c**) T cells in lymphocytes between the metformin treatment and control. Expressed as the mean ± SEM. *P < 0.05, **P < 0.01; Mann–Whitney test (two-tailed) and paired Student’s t-test. ELISA, Enzyme-linked immunosorbent assay. Met (-), control; Met ( +),20 mM metformin treatment

Telomere, a DNA–protein complex located at the end of chromosomes, plays a crucial role in maintaining chromosome stability. However, during cell divisions, the length of telomeres naturally decreases, ultimately leading to cell aging, death, or cancer [8]. Telomerase, an enzyme with reverse transcription activity, is responsible for synthesizing telomeric DNA and preserving the length of telomeres [8]. As T cells age, telomeres shorten and telomerase activity decreases, resulting in the loss of their proliferative ability [8]. Our above results indicate that metformin is able to reduce the number of senescent T cells, whether it has an effect on telomerase is still unclear. We collected the supernatant of PBMCs that were cultured with 20 mM metformin for 24 h in the middle-aged group and measured telomerase concentration using an enzyme-linked immunosorbent assay (ELISA). The results showed that the telomerase concentration in the control group was 0.71 ng/ml (0.71 ± 0.07, *N* = 28). However, with metformin treatment, the concentration of telomerase increased to 1.33 ng/ml (1.33 ± 0.24 *N* = 28) (*P* < 0.05, Fig. 7a). Immunosenescence is characterized by an increase in aged T cells and a decrease in naive T cells which possess stemness [10]. Consistent with previous studies, our results also revealed a lower proportion of CD8 + Tn (CD3 + CD8 + CD45RA + CCR7 +) cells in the elderly group (3%) compared to the middle-aged group (6%) and the young group (9%) (*P* < 0.05, Supplementary Fig. 6). Surprisingly, metformin increased the frequency of undifferentiated T cells (CD3 + CD4 + /CD8 + CD45RA + CCR7 + CD27 + CD28 +) (*P* < 0.05, Fig. 7b, c). These findings suggest that the increased telomerase concentration and number of undifferentiated T cells induced by metformin may contribute to its anti-aging effects.

### Metformin promoted the expression of genes related to stemness and telomerase activity, inhibited the expression of β-galactosidase (SA-β-Gal) and DNA damage associated genes

To provide a detailed characterization of cellular senescence, including senescence-associated β-galactosidase (SA-β-Gal) production, telomere shortening, decreased telomerase activity, and increased DNA damage, cell cycle arrest [12, 30], we conducted RNA-sequencing on PBMCs obtained from three middle-aged subjects. The cells were treated with metformin or served as control groups to examine the effects.

**Fig. 8:**
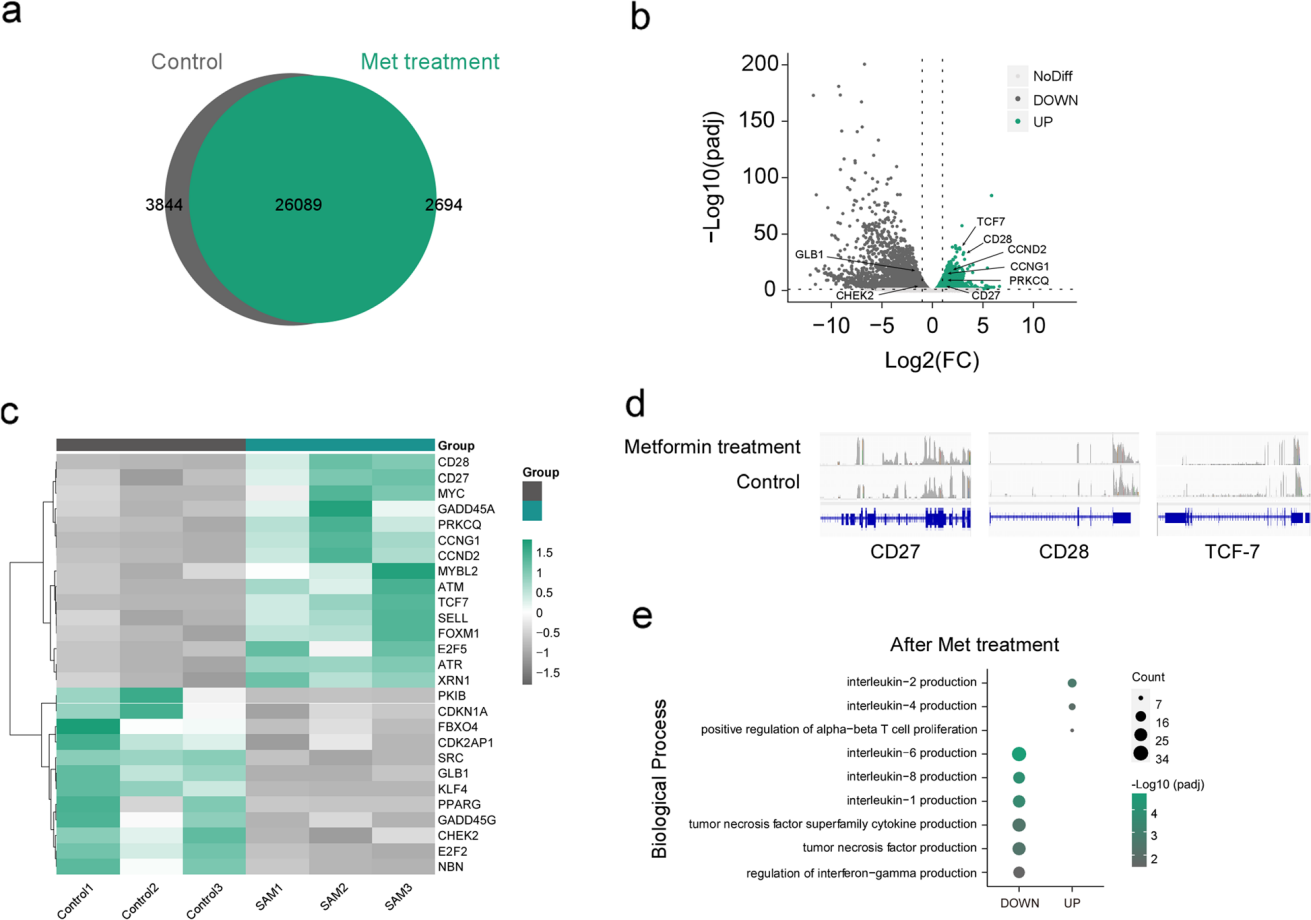
The RNA -seq analysis of gene expression associated with T cell senescence with metformin treatment. **a** Venn diagram portraying the intersections and disjunctive unions of differentially expressed genes in metformin treatment group (green) and control group (gray). **b** The Volcano Plot showed the overall distribution of genes with up-regulated expression (green), down-regulated expression (dark gray) and no significant difference (light gray) in the metformin treatment group compared to the control group. **c** Expression heatmap of a selection of senescence- and stemness-associated genes in PBMCs from metformin treatment group and control group at middle-age subjects (*n* = 3). Data are represented as Z-scores. **d** Representative genome browser visualizations of normalized reads at indicated gene loci in PBMCs from a representative donor before and after metformin treatment. **e** The chart portrayed a selection of biological process, from GO functional enrichment analysis, which were up-regulated and down-regulated with metformin treatment. All the above RNA sequencing datas came from three independent donors and three independent experiments

There were 26,089 differentially expressed genes (DEGs) co-expressed between the group treated with metformin and the control group; additionally, there were 2,694 specifically expressed DEGs in the metformin treatment group and 3,844 in the control group (Fig. 8a). To characterize the transcriptomic changes between metformin treatment and the control group, DEGs were displayed using a volcano plot (Fig. 8b) and expression heatmap (Fig. 8c). The gene GLB1, which is associated with SA-β-Gal, was significantly decreased in the metformin-treated group compared with the control group (Fig. 8b, c). DNA damage-associated genes, including CDKN1A, CHEK2, E2F2, NBN, and GADD45G [31–34], were downregulated with metformin treatment (Fig. 8c). In contrast, metformin promoted the expression of cyclin-related genes CCNG1 and CCND2 (Fig. 8b, c). Consistent with ELISA data from telomerase studies, the expression of ATM, ATR [35], PRKCQ [36], and MYC [10] mRNA was significantly elevated in the metformin treatment group (Fig. 8c). DEGs revealed increased expression of TCF7, SELL, CD28, CD27 [37], genes associated with stemness (Fig. 8b-d), which is consistent with flow cytometry analysis where the frequencies of undifferentiated T cells were significantly increased in the metformin treatment group (Fig. 7b, c). A detailed analysis of the DEGs was performed by the functional annotation of Gene Ontology (GO), which showed enrichment for a highly diverse array of biological processes consistent with the change of cytokine secretion with metformin treatment. Biological processes related to lymphocyte proliferation were upregulated, such as interleukin-2 (IL-2) and interleukin-4 (IL-4); however, SASP-associated biological processes, such as IL-6, IL-8, IL-1, TNF, and IFN-γ production, were decreased with metformin treatment (Fig. 8e). These results supported that metformin promoted the expression of genes related to stemness and telomerase activity, but inhibited the expression of cellular senescence-related genes.

## Discussion

The aim of this study is to explore the inhibition of cellular senescence in T cells with metformin treatment. We examined the frequency of senescent T cells across different age groups, and observed that the middle-aged group (45–65 years old) had a higher number of senescent T cells compared to the elderly group (> 65 years old), which has not been previously reported. Our findings are inconsistent with the proposal that the number of senescent cells increases with age [38]. Whether the highest proportion of aged T cells in the middle-aged group leads to the decline of immune function requires further examination. Similarly, we observed that the incidence of lung cancer was the highest in the middle-aged group. This could be related to the higher number of senescent T cells in this group. Accumulating evidence suggests that senescent T cells are associated with promotion of tumorigenesis and progression [39]. The elevated levels of SASP by senescent T cells, such as IL-6, IL-8, and IL-18, promote tumor development and suppress antitumor immunity, regulating the tumor microenvironment through proinflammatory and anti-inflammatory cytokines [40].

It has been reported that metformin has anti-aging and anti-inflammatory properties. In our study, we treated PBMCs from the middle age group with metformin. Our findings demonstrated that metformin could decrease the frequency of CD8 + senescent T cells and affect the secretion of SASP, metformin inhibits the secretion of IFN-γ in CD8 + senescent T cells, suggesting its potential role in alleviating chronic inflammation and SASP production by aged T cells [41]. Importantly, metformin has no effect on the IFN-γ production of non-senescent T cells, indicating its ability to preserve the anti-tumor and anti-viral effects of T cells. However, the specific mechanism of action remains unclear and requires further investigation. Additionally, Interleukin-6 (IL-6) is acknowledged as one of the most versatile cytokines. IL-6 is involved in immunomodulatory responses and the development, proliferation, and differentiation of multiple cell types [26]. It is believed that IL-6 promotes the occurrence and development of tumors as a proinflammatory cytokine [42]. IL-6 is mainly secreted by innate immune cells and is produced less frequently in T cells. Our results showed that metformin reduces IL-6 production in both CD3 negative cells and T cells in PBMCs and thus reduce the source of SASP. TNF-α is involved in the maintenance and homeostasis of the immune system, inflammation, and host defense [28, 29]. However, in the elderly, TNF-α can participate in a variety of pathological processes, such as inflammaging, autoimmunity, and cancer [43]. According to our results, metformin intervention promoted the expression of TNF-α in both aged T cell subsets and non-aged T cell subsets. This may be a side effect of metformin, and attention should be paid to the different responses of individuals during its clinical application as an anti-aging treatment.

The inability of T cells to proliferate is partially caused by telomere erosion and loss of telomerase activity. The length of telomeres will be shortened with an increase in the count of cell divisions, guaranteeing the stable and intact presence of DNA and genetic information in cells [8]. With increasing age, senescent T cells gradually accumulate, and their telomeres shorten, causing a decrease in telomerase activity and a loss of their proliferative ability [8]. Additionally, the expression of costimulatory receptors CD27 and CD28 also declines in senescent T cells. It has been demonstrated that the CD28 costimulatory signal plays a crucial role in telomerase induction in human T cells, and the loss of these surface markers may result in decreased telomerase activity [44]. Our results suggest that metformin increases the concentration of telomerase and the frequency of undifferentiated T cells (CD3 + CD4 + /CD8 + CD45RA + CCR7 + CD27 + CD28 +), which may mediate the anti-aging effects. A limitation of this study is that the length of telomeres in aged T cells with metformin treatment was not examined due to the limited number of T cells. The relationship between the increased concentration of telomerase and telomerase activity should be confirmed in future research. RNA sequencing results show a decrease in the expression of SA-β-Gal associated genes with metformin treatment, and future studies will be performed at the protein level. Further studies will be conducted to investigate the effect of metformin on the energy metabolism of senescent T cells.

## Conclusions

In this study, we present evidence that metformin exerts an anti-aging effect on senescent T cells. Immunosenescence, especially the senescence of T cells, can lead to the dysfunction of immune function and the occurrence of aging diseases. We found that the middle-aged group had more senescent T cells than the elderly group; Metformin plays an anti-aging role by reducing the number of senescent T cells, reducing the production of SASP, and promoting the increase of telomerase and the number of stem T cells. The significance of our study is to expand the scope of clinical application of metformin by inhibiting immune senescence, and to provide theoretical support for its existing clinical applications.

## Materials and methods

### Ethics statement

Written informed consent was obtained from all donors. Ethics approval for this study was obtained from the ethics committees of the Zhong Shan Medical School, Sun Yat-San University (Guangzhou, China) and First Affiliated Hospital of Sun Yat-San University (Guangzhou, China).

### Study participants

In total, 88 healthy donors were enrolled from the First Affiliated Hospital of Sun Yet-San University of Guang Zhou, China, divided into three group: 26 young donors (mean age = 34.1), 34 middle-aged donors (mean age = 54.13), and 28 elderly donors (mean age = 75.57). All donors, recruited for blood collection, were HIV negative, HBV negative, HCV negative and free of cancer and other infection diseases. Four thousand four hundred ninety-eight lung cancer cases from Sun Yat-sen University First Affiliated Hospital over the past six years (2017–2023) were used for Clinical retrospective analysis.

### Preparation of PBMCs

PBMCs were isolated by Ficoll-Hypaque (Tian Jin Hao Yang Biological Manufacture, China; cat.LTS1077) gradient centrifugation of sodium heparin-blood obtained from HDs. PBMCs were resuspended in complete RPMI 1640 medium (GIBCO, cat.C11875500BT) supplemented with 10% Foetal Bovine Serum (Biological Industries, cat.04–011-1ACS), 1% Penicillin–Streptomycin (GIBCO, cat.15140–122).

### Flow cytometry

#### Phenotypic characterization

PBMCs which cultured with 0 mmol, 5 mmol, 10 mmol or 20 mmol metformin (GLPBIO, cat. GC60245) at 37℃ for 24 h were stained for phenotypic analyses. The following panel of mouse anti-human mAbs was used: APC.cy7-conjugated anti-CD3 (TONBO biosciences, 25–0038-T100), Percp.cy5.5-conjugated anti-CD8 (TONBO biosciences, 65–0088-7100), PE.Cyanine7-conjugated anti-CD8 (BD Pharmingen, 557746), Alexa Fluor 700-conjugated anti-CD45RA (BD Pharmingen, 560673), Brilliant Violet 510-conjugated anti-CCR7 (Biolegend, 353232), FITC-conjugated anti-CD28 (TONBO biosciences, 35–0289-T025), PE-conjugated anti-CD28 (Biolegend, 302907), APC-conjugated anti-CD27 (eBioscience, 17–0279-42), Percp.cy5.5-conjugated anti-CD27 (BD biosciences, 560612), Pacific Blue-conjugated anti-CD57 (Biolegend, 359607), Super Bright 600-conjugated anti-KLRG1 (eBioscience, 63–9488-42).

#### Cytokines staining

After metformin treatment, PBMCs were incubated at 2 × 10^6^ cells per well in RP10 media (RPMI, 10% Foetal Bovine Serum, 1% Penicillin–Streptomycin) alone or with 1X Cell Stimulation Cocktail (TONBO biosciences, cat.TNB-4975-UL100) for 4–6 h at 37℃. Cells were then harvested and intracellular stained according to the protocol of eBioscience Intracellular Fixation & Permeabilization Buffer Set (eBioscience, cat.88–8824-00). The following panel of mouse anti-human mAbs was used: APC-conjugated anti-IFN-γ (BD Pharmingen, 554702), APC-conjugated anti-TNF-α (eBioscience, 17–7349-82), PE-conjugated anti-Granzyme B (eBioscience, 12–8899-41), PE-conjugated anti-IL-6 (Biolegend, 501106). All samples were collected on FACSAria II BD (Mountain View, CA, USA). Data were analyzed using Flow Jo software (Tree Star, San Carlos, CA, USA).

#### Human telomerase enzyme linked immunosorbent assay

A commercial ELISA kit (ELK Biotechnology, cat. ELK8668) was used to test the telomerase concentration in medium of PBMCs with metformin treatment or control. The assay procedures were performed according to the manufacturer’s instructions. The concentration of telomerase was determined by the mean optical density of 450 nm, and a standard curve was fitted with Polynomial Curve Fitting.

#### RNA-seq

The quality and purity of RNA from middle-age donors (*n* = 3) were examined by a NanoDrop™ One/OneC spectrophotometer (Thermo Scientific, Waltham, MA, USA) and Life Invitrogen Qubit RNA BR (Broad-Range) Assay Kit. RNA integrity was analyzed using Agilent 4200 TapeStation system (Agilent, Santa Clara, CA, USA). The NEBNext® Poly(A) mRNA Magnetic Isolation Module and NEBNext® Ultra™ II mRNA Library Prep Kit for Illumina® were used for mRNA isolation and library construction following the manufacturers’ protocols. High-throughput transcriptome sequencing was performed on an Illumina NovaSeq 6000 platform according to the manufacturer’s instructions. FASTQ files were aligned to the version 19 of the human reference genome (hg19) using HISAT2. Reads per exon (hg19) were quantified using HTseq. Differential expression analysis was performed with DESeq2 and edegR. ClusterProfier was used for gene functional enrichment annotation.

### Statistical analysis

Statistical analysis was performed on the GraphPad Prism software version 5. The Mann–Whitney test (two-tailed) and non-paired Student’s t-test or paired Student’s t-test were performed to determine the statistical differences in the two groups. A value of *P* < 0.05 was considered statistically significant.

### Supplementary Information

**Additional file 1: Supplementary Figure 1. **The composition of T cell subsets at different age groups. (a)The proportion of lung cancer patients at different ages from Sun Yat-sen University First Affiliated Hospital from 2017-2023 (*n*=4498). The frequency of senescent T cells (b), Teff cells (CD3+CD45RA+CCR7-) (d) and Tem cells (CD3+CD45RO+CCR7-) (e) in CD8+T cells and CD4+T cells at different age groups. (c) Pie charts depicting the events of Tn (CD3+CD45RA+CCR7+), Tcm (CD3+CD45RO+CCR7+), Tem and Teff cell subsets of CD8+ and CD4+T cells from different age groups. Expressed as the mean ± SEM. *P < 0.05, **P < 0.01, ***P < 0.001; Mann– Whitney test (two-tailed) and nonpaired Student’s t-test. Tn, naïve T cell; Teff, effector T cell; Tem, effector memory T cell; Tcm, central memory T cell. Young, young-age group; Middle, middle-age group; old, elderly group.

**Additional file 2: Supplementary Figure 2.** The different concentration of metformin treatment on PBMCs from middle-aged group. The frequency of CD27-CD28-CD57+KLRG1+T cells from Teff (CD3+CD8+CD45RA+CCR7-) (a) and Tem (CD3+CD8+CD45RO+CCR7-) (b), treated with 0mM, 5mM or 10mM for 24 hours at middle age group. Expressed as the mean ± SEM. ***P < 0.001; paired *t* test. Teff, effector T cells; Tem, effector memory T cells.

**Additional file 3: Supplementary Figure 3.** The effect on IFN-γ production in CD4+senescent T cells with metformin treatment. Representative flow histogram of IFN-γ production by CD4+ senescent T cells (CD3+CD4+CD45RA+CCR7-CD27-CD28-CD57+KLRG1+) (a) and non- senescent T cells (CD3+CD4+CD45RA+CCR7-CD27-CD28-CD57-KLRG1-) (b) between the control and the 20mM Metformin treatment group relative to the unstimulated controls from middle-age donors. Quantification of the frequency of IFN-γ-expressing cells and the plot of IFN-γ-MFI in senescent T cells (a) and non-senescent T cell population (b) from CD4+T cells. Expressed as the mean ± SEM. **P < 0.01; Paired t test. Met, metformin.

**Additional file 4: Supplementary Figure 4.** IL-6 secretion in CD8+ and CD4+T cells was inhibited by Metformin. Analysis of IL-6 production in CD8+T cells (a) and CD4+T cells (b), PBMCs cultured with 0 or 20mM Met for 24 hours in middle age group. Expressed as the mean ± SEM. *P < 0.05, ***P < 0.001; Paired *t* test. Met, metformin.

**Additional file 5:** **Supplementary Figure 5.** The effect of metformin treatment on the production of TNF-a in CD4+senescent T cells. Analysis of TNF-α-secreting from CD4+senescence T cells (CD3+CD4+CD45RA+CCR7-CD27-CD28-CD57+KLRG1+) (a) and CD3+CD4+CD45RA+CCR7-CD27-CD28-CD57-KLRG1-T cells (b) with 20mM metformin treatment. (c)The frequency of CD3+CD4+CD45RA+CCR7-CD27-CD28-CD57-KLRG1-TNF-α+ in lymphocytes with or not metformin treatment. Expressed as the mean ± SEM. *P < 0.05; Paired *t *test. Met, metformin.

**Additional file 6: Supplementary Figure 6.** The distribution of naïve T cells (CD3+CD4+/CD8+CD45RA+CCR7+) at different age groups. Expressed as the mean ± SEM. *P < 0.05, ***P < 0.001; Mann–Whitney test (two-tailed) and unpaired Student’s t-test. Young, young age group; Middle, middle age group; Old, elderly group; Tn, naïve T cell.

## Data Availability

All data generated or analysed during this study are included in this published article.

## References

[1] Rodriguez IJ (2020). N Lalinde Ruiz, M Llano León, L Martínez Enríquez, MDP Montilla Velásquez, JP Ortiz Aguirre, OM Rodríguez Bohórquez, EA Velandia Vargas, ED Hernández, CA Parra López, Immunosenescence Study of T Cells: A Systematic Review. Frontiers in immunology.

[2] Pangrazzi L, Weinberger B (2020). T cells, aging and senescence. Exp Gerontol.

[3] Zhang J, He T, Xue L, Guo H (2021). Senescent T cells: a potential biomarker and target for cancer therapy. EBioMedicine.

[4] Akbar AN, Henson SM, Lanna A (2016). Senescence of T lymphocytes: implications for enhancing human immunity. Trends Immunol.

[5] Carrasco E, Gómez de Las Heras MM, Gabandé-Rodríguez E, Desdín-Micó G, Aranda JF, Mittelbrunn M (2022). The role of T cells in age-related diseases nature reviews. Immunology.

[6] Henson SM, Macaulay R, Riddell NE, Nunn CJ, Akbar AN (2015). Blockade of PD-1 or p38 MAP kinase signaling enhances senescent human CD8(+) T-cell proliferation by distinct pathways. Eur J Immunol.

[7] Callender LA, Carroll EC, Beal RWJ, Chambers ES, Nourshargh S, Akbar AN, Henson SM (2018). Human CD8(+) EMRA T cells display a senescence-associated secretory phenotype regulated by p38 MAPK. Aging Cell.

[8] Chakravarti D, LaBella KA, DePinho RA (2021). Telomeres: history, health, and hallmarks of aging. Cell.

[9] López-Otín C, Blasco MA, Partridge L, Serrano M, Kroemer G (2013). The hallmarks of aging. Cell.

[10] Khattar E, Tergaonkar V (2017). Transcriptional Regulation of Telomerase Reverse Transcriptase (TERT) by MYC. Front Cell Dev Biol.

[11] Libri V, Azevedo RI, Jackson SE, Di Mitri D, Lachmann R, Fuhrmann S, Vukmanovic-Stejic M, Yong K, Battistini L, Kern F, Soares MV, Akbar AN (2011). Cytomegalovirus infection induces the accumulation of short-lived, multifunctional CD4+CD45RA+CD27+ T cells: the potential involvement of interleukin-7 in this process. Immunology.

[12] Liu X, Mo W, Ye J, Li L, Zhang Y, Hsueh EC, Hoft DF, Peng G (2018). Regulatory T cells trigger effector T cell DNA damage and senescence caused by metabolic competition. Nat Commun.

[13] Nojima I, Wada J (2023). Metformin and Its Immune-Mediated Effects in Various Diseases. Int J Mol Sci.

[14] Chen S, Gan D, Lin S, Zhong Y, Chen M, Zou X, Shao Z, Xiao G (2022). Metformin in aging and aging-related diseases: clinical applications and relevant mechanisms. Theranostics.

[15] Moiseeva O, Deschênes-Simard X, St-Germain E, Igelmann S, Huot G, Cadar AE, Bourdeau V, Pollak MN, Ferbeyre G (2013). Metformin inhibits the senescence-associated secretory phenotype by interfering with IKK/NF-κB activation. Aging Cell.

[16] Childs BG, Gluscevic M, Baker DJ, Laberge RM, Marquess D, Dananberg J, van Deursen JM (2017). Senescent cells: an emerging target for diseases of ageing. Nat Rev Drug Discovery.

[17] Collin SM, Bakken IJ, Nazareth I, Crawley E, White PD (2017). Trends in the incidence of chronic fatigue syndrome and fibromyalgia in the UK, 2001–2013: a Clinical Practice Research Datalink study. J R Soc Med.

[18] Maassen JM, Bergstra SA, Chopra A, Govind N, Murphy EA, Vega-Morales D, Huizinga TWJ, Allaart CF (2021). Phenotype and treatment of elderly onset compared with younger onset rheumatoid arthritis patients in international daily practice. Rheumatology (Oxford).

[19] Kumar BV, Connors TJ, Farber DL (2018). Human T Cell Development, localization, and function throughout Life. Immunity.

[20] Ando M, Ito M, Srirat T, Kondo T, Yoshimura A (2020). Memory T cell, exhaustion, and tumor immunity. Immunological medicine.

[21] Ren J, Zhang Y (2018). Targeting Autophagy in Aging and Aging-Related Cardiovascular Diseases. Trends Pharmacol Sci.

[22] Foretz M, Guigas B, Viollet B. Understanding the glucoregulatory mechanisms of metformin in type 2 diabetes mellitus. Nat Rev Endocrinol. 2019;15(10):569–89. 10.1038/s41574-019-0242-2. Epub 2019 Aug 22. 10.1038/s41574-019-0242-2 31439934

[23] Kumari R, Jat P (2021). Mechanisms of cellular senescence: cell cycle arrest and senescence associated secretory phenotype. Fronti Cell Dev Biol.

[24] Xu W, Larbi A (2017). Markers of T Cell Senescence in Humans. Int J Mol Sci.

[25] Khosla S, Farr JN, Tchkonia T, Kirkland JL (2020). The role of cellular senescence in ageing and endocrine disease. Nat Rev Endocrinol.

[26] Tanaka T, Narazaki M, Kishimoto T (2014). IL-6 in inflammation, immunity, and disease. Cold Spring Harb Perspect Biol.

[27] Kared H, Martelli S, Ng TP, Pender SL, Larbi A (2016). CD57 in human natural killer cells and T-lymphocytes. Cancer Immunol Immunother.

[28] Zelová H, Hošek J (2013). TNF-α signalling and inflammation: interactions between old acquaintances. Inflamm Res.

[29] Mehta AK, Gracias DT, Croft M (2018). TNF activity and T cells. Cytokine.

[30] Martínez-Zamudio RI, Dewald HK, Vasilopoulos T, Gittens-Williams L, Fitzgerald-Bocarsly P, Herbig U (2021). Senescence-associated β-galactosidase reveals the abundance of senescent CD8+ T cells in aging humans. Aging Cell.

[31] Lulli M, Del Coco L, Mello T, Sukowati C, Madiai S, Gragnani L, Forte P, Fanizzi FP, Mazzocca A, Rombouts K, Galli A, Carloni V (2021). DNA damage response protein CHK2 regulates metabolism in liver cancer. Can Res.

[32] Ou HL, Schumacher B (2018). DNA damage responses and p53 in the aging process. Blood.

[33] Pennisi R, Antoccia A, Leone S, Ascenzi P, di Masi A (2017). Hsp90α regulates ATM and NBN functions in sensing and repair of DNA double-strand breaks. FEBS J.

[34] Castillo DS, Campalans A, Belluscio LM, Carcagno AL, Radicella JP, Cánepa ET, Pregi N (2015). E2F1 and E2F2 induction in response to DNA damage preserves genomic stability in neuronal cells. Cell Cycle.

[35] Tong AS, Stern JL, Sfeir A, Kartawinata M, de Lange T, Zhu XD, Bryan TM (2015). ATM and ATR signaling regulate the recruitment of human telomerase to telomeres. Cell Rep.

[36] Kim YW, Hur SY, Kim TE, Lee JM, Namkoong SE, Ki IK, Kim JW (2001). Protein kinase C modulates telomerase activity in human cervical cancer cells. Exp Mol Med.

[37] Galletti G, De Simone G, Mazza EMC, Puccio S, Mezzanotte C, Bi TM, Davydov AN, Metsger M, Scamardella E, Alvisi G, De Paoli F, Zanon V, Scarpa A, Camisa B, Colombo FS, Anselmo A, Peano C, Polletti S, Mavilio D, Gattinoni L, Boi SK, Youngblood BA, Jones RE, Baird DM, Gostick E, Llewellyn-Lacey S, Ladell K, Price DA, Chudakov DM, Newell EW, Casucci M, Lugli E (2020). Two subsets of stem-like CD8(+) memory T cell progenitors with distinct fate commitments in humans. Nat Immunol.

[38] Goronzy JJ, Weyand CM (2017). Successful and Maladaptive T Cell Aging. Immunity.

[39] Ye J, Peng G (2015). Controlling T cell senescence in the tumor microenvironment for tumor immunotherapy. Oncoimmunology.

[40] Tchkonia T, Zhu Y, van Deursen J, Campisi J, Kirkland JL (2013). Cellular senescence and the senescent secretory phenotype: therapeutic opportunities. J Clin Investig.

[41] Jorgovanovic D, Song M, Wang L, Zhang Y (2020). Roles of IFN-γ in tumor progression and regression: a review. Biomarker research.

[42] Jones SA, Jenkins BJ (2018). Recent insights into targeting the IL-6 cytokine family in inflammatory diseases and cancer. Nat Rev Immunol.

[43] Hayden MS, Ghosh S (2014). Regulation of NF-κB by TNF family cytokines. Semin Immunol.

[44] Akbar AN, Beverley PC, Salmon M (2004). Will telomere erosion lead to a loss of T-cell memory?. Nat Rev Immunol.

