# Supplementary figures and images for: The effect of metformin on senescence of T lymphocytes
Source: Immun Ageing. 2023 Dec 12;20:73. doi: 10.1186/s12979-023-00394-0 (PMC10714529; doi:10.1186/s12979-023-00394-0)

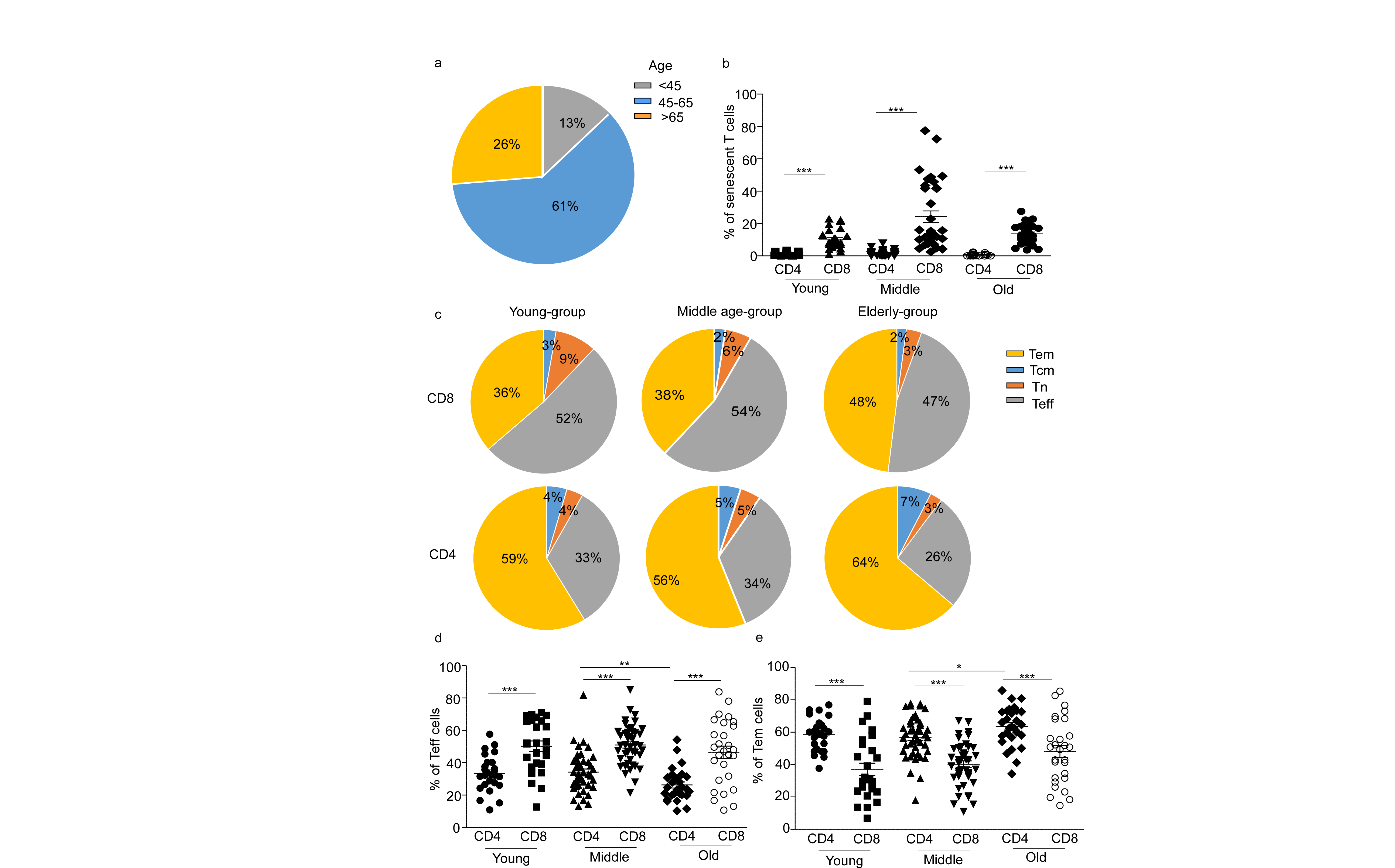

Supplement: Supplementary file 1 — Additional file 1: Supplementary Figure 1. The composition of T cell subsets at different age groups. (a)The proportion of lung cancer patients at different ages from Sun Yat-sen University First Affiliated Hospital from 2017-2023 (n=4498). The frequency of senescent T cells (b), Teff cells (CD3+CD45RA+CCR7-) (d) and Tem cells (CD3+CD45RO+CCR7-) (e) in CD8+T cells and CD4+T cells at different age groups. (c) Pie charts depicting the events of Tn (CD3+CD45RA+CCR7+), Tcm (CD3+CD45RO+CCR7+), Tem and Teff cell subsets of CD8+ and CD4+T cells from different age groups. Expressed as the mean ± SEM. *P < 0.05, **P < 0.01, ***P < 0.001; Mann– Whitney test (two-tailed) and nonpaired Student’s t-test. Tn, naïve T cell; Teff, effector T cell; Tem, effector memory T cell; Tcm, central memory T cell. Young, young-age group; Middle, middle-age group; old, elderly group. [file 12979_2023_394_MOESM1_ESM.tif]

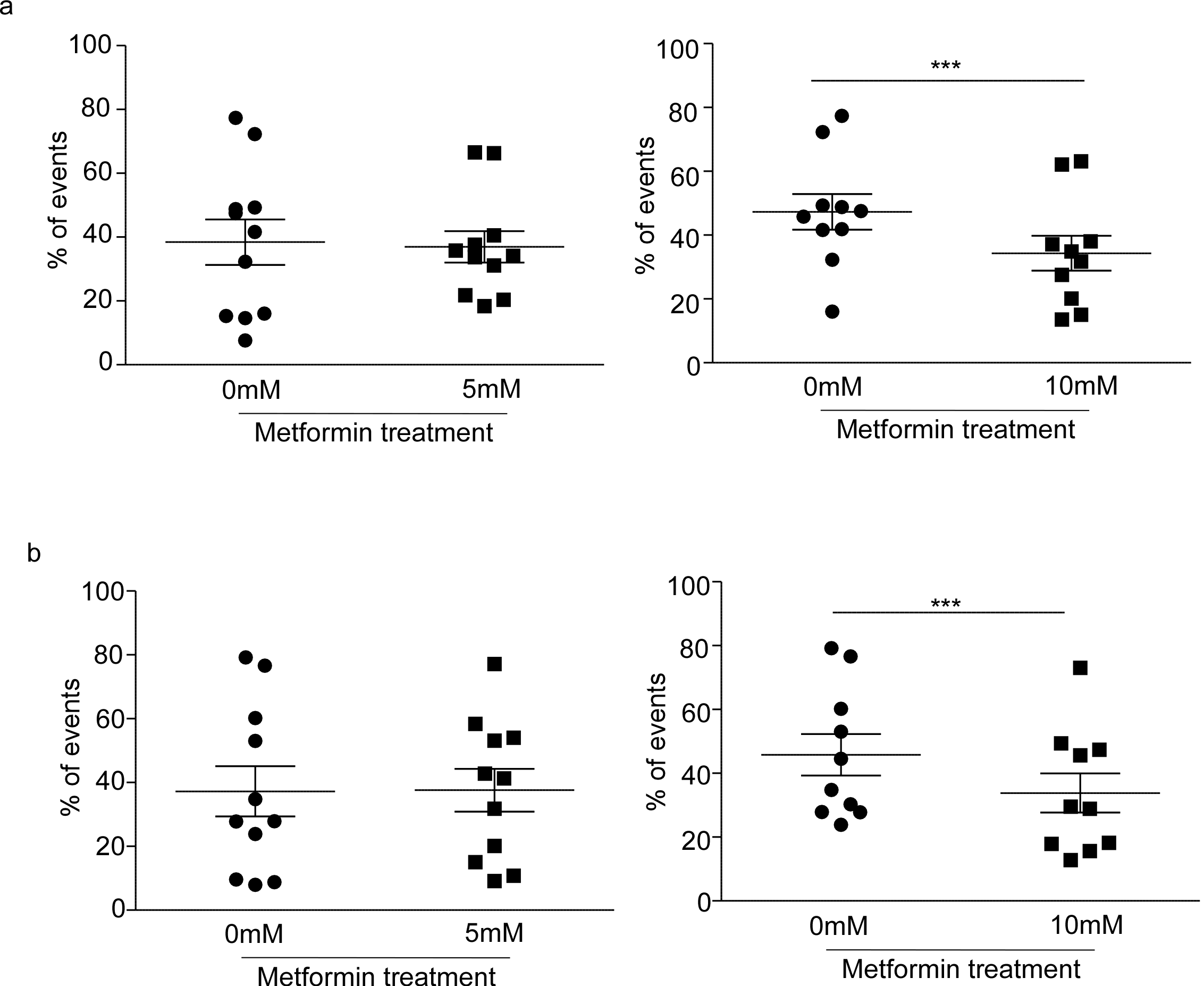

Supplement: Supplementary file 2 — Additional file 2: Supplementary Figure 2. The different concentration of metformin treatment on PBMCs from middle-aged group. The frequency of CD27-CD28-CD57+KLRG1+T cells from Teff (CD3+CD8+CD45RA+CCR7-) (a) and Tem (CD3+CD8+CD45RO+CCR7-) (b), treated with 0mM, 5mM or 10mM for 24 hours at middle age group. Expressed as the mean ± SEM. ***P < 0.001; paired t test. Teff, effector T cells; Tem, effector memory T cells. [file 12979_2023_394_MOESM2_ESM.tif]

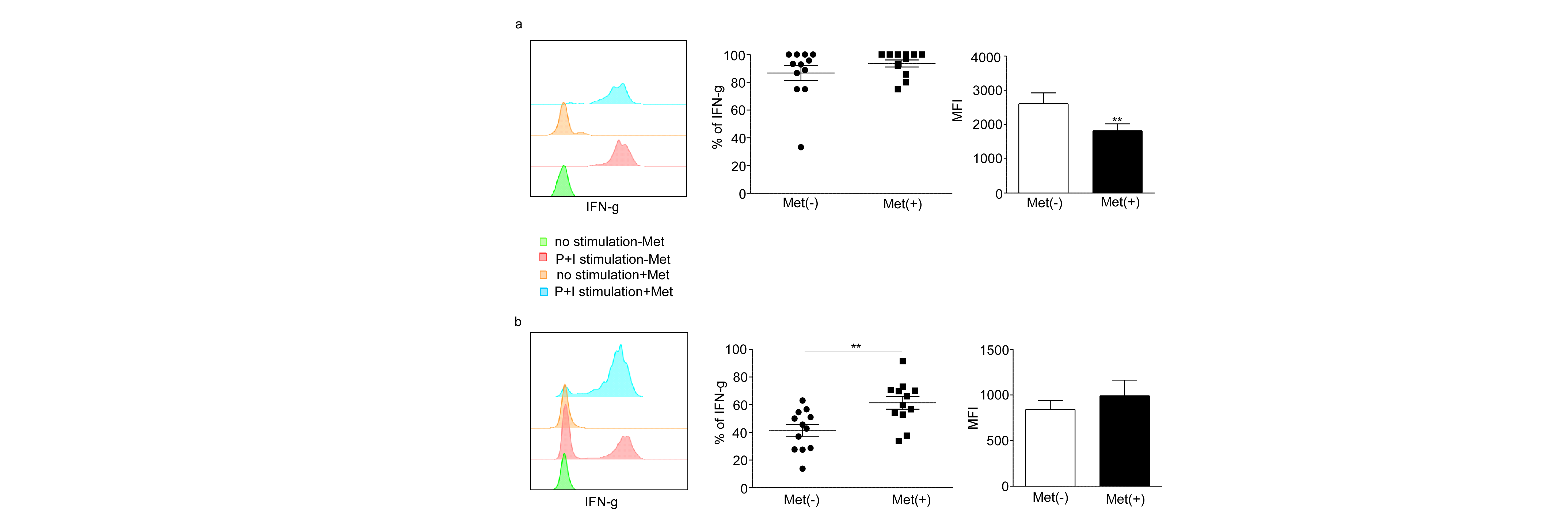

Supplement: Supplementary file 3 — Additional file 3: Supplementary Figure 3. The effect on IFN-γ production in CD4+senescent T cells with metformin treatment. Representative flow histogram of IFN-γ production by CD4+ senescent T cells (CD3+CD4+CD45RA+CCR7-CD27-CD28-CD57+KLRG1+) (a) and non- senescent T cells (CD3+CD4+CD45RA+CCR7-CD27-CD28-CD57-KLRG1-) (b) between the control and the 20mM Metformin treatment group relative to the unstimulated controls from middle-age donors. Quantification of the frequency of IFN-γ-expressing cells and the plot of IFN-γ-MFI in senescent T cells (a) and non-senescent T cell population (b) from CD4+T cells. Expressed as the mean ± SEM. **P < 0.01; Paired t test. Met, metformin. [file 12979_2023_394_MOESM3_ESM.tif]

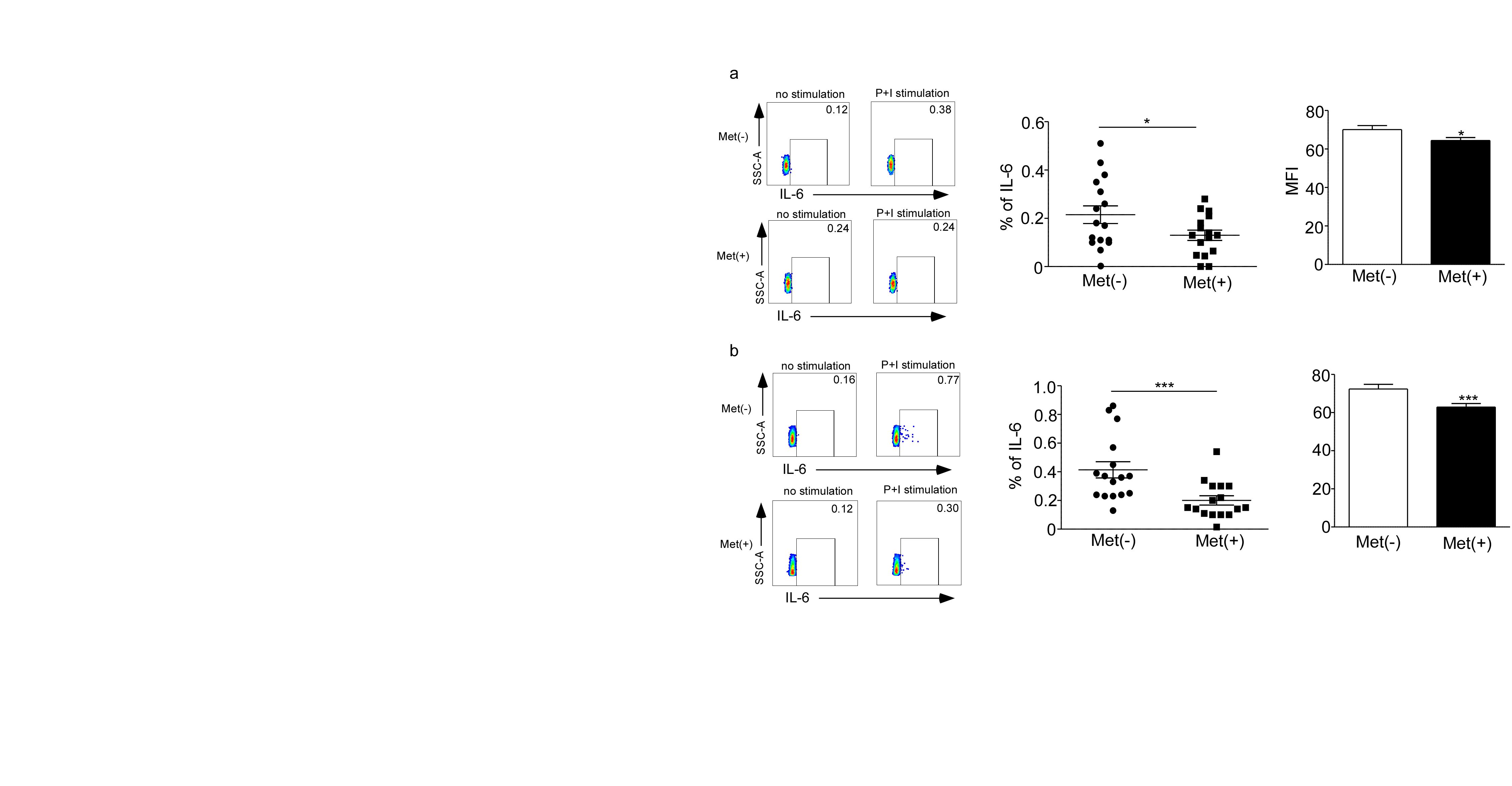

Supplement: Supplementary file 4 — Additional file 4: Supplementary Figure 4. IL-6 secretion in CD8+ and CD4+T cells was inhibited by Metformin. Analysis of IL-6 production in CD8+T cells (a) and CD4+T cells (b), PBMCs cultured with 0 or 20mM Met for 24 hours in middle age group. Expressed as the mean ± SEM. *P < 0.05, ***P < 0.001; Paired t test. Met, metformin. [file 12979_2023_394_MOESM4_ESM.tif]

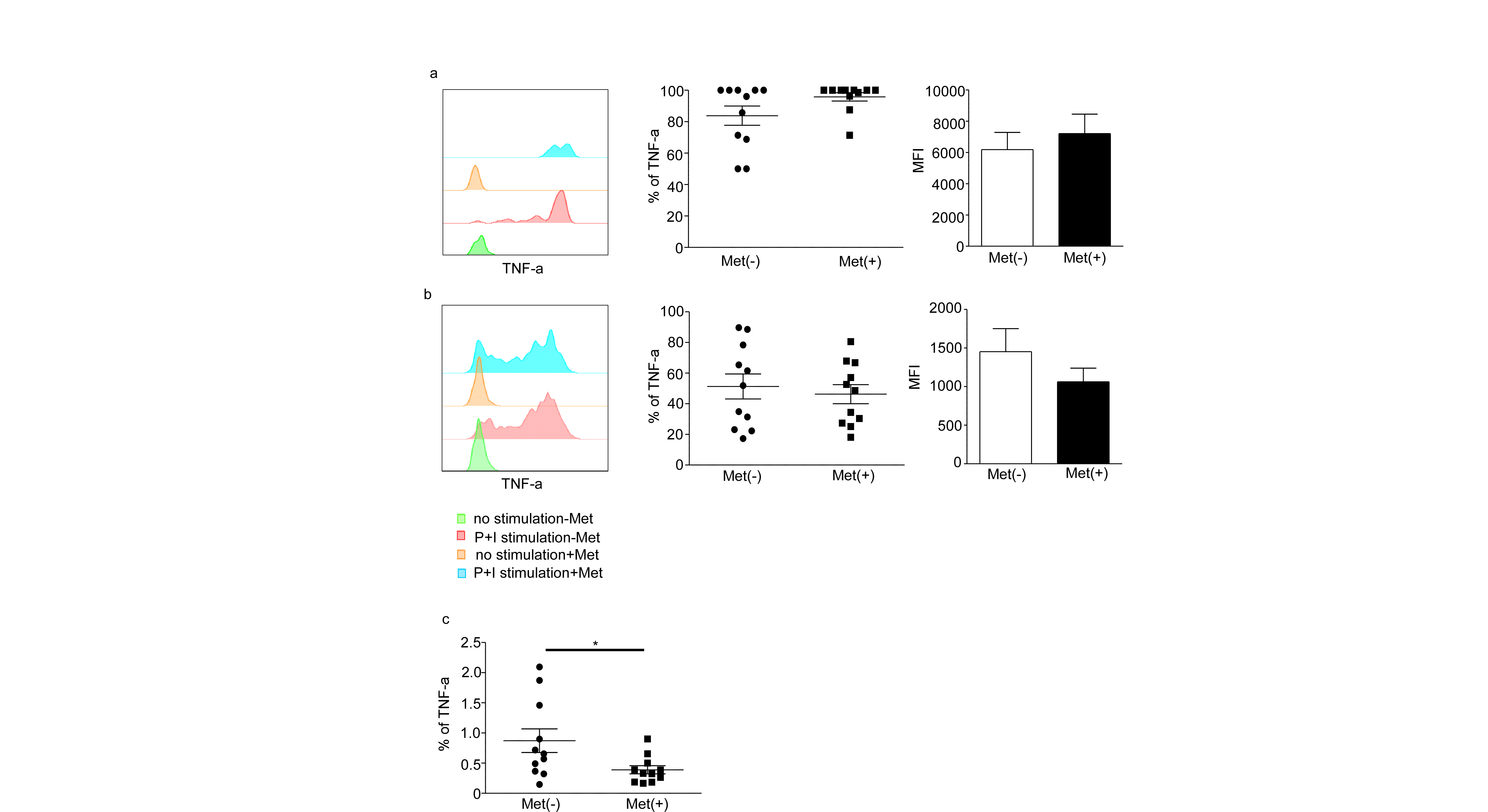

Supplement: Supplementary file 5 — Additional file 5: Supplementary Figure 5. The effect of metformin treatment on the production of TNF-a in CD4+senescent T cells. Analysis of TNF-α-secreting from CD4+senescence T cells (CD3+CD4+CD45RA+CCR7-CD27-CD28-CD57+KLRG1+) (a) and CD3+CD4+CD45RA+CCR7-CD27-CD28-CD57-KLRG1-T cells (b) with 20mM metformin treatment. (c)The frequency of CD3+CD4+CD45RA+CCR7-CD27-CD28-CD57-KLRG1-TNF-α+ in lymphocytes with or not metformin treatment. Expressed as the mean ± SEM. *P < 0.05; Paired t test. Met, metformin. [file 12979_2023_394_MOESM5_ESM.tif]

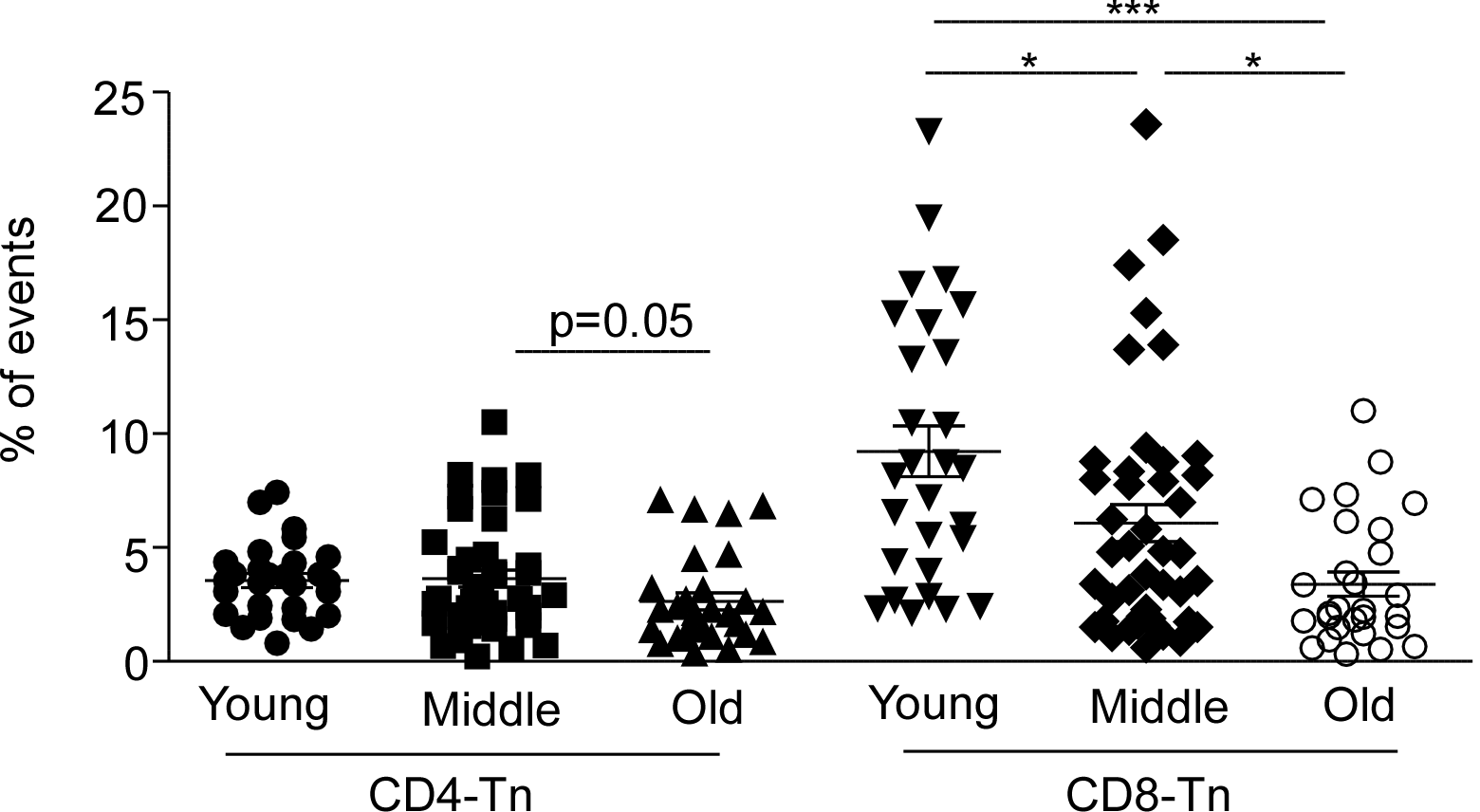

Supplement: Supplementary file 6 — Additional file 6: Supplementary Figure 6. The distribution of naïve T cells (CD3+CD4+/CD8+CD45RA+CCR7+) at different age groups. Expressed as the mean ± SEM. *P < 0.05, ***P < 0.001; Mann–Whitney test (two-tailed) and unpaired Student’s t-test. Young, young age group; Middle, middle age group; Old, elderly group; Tn, naïve T cell. [file 12979_2023_394_MOESM6_ESM.tif]
